# Core genome multilocus sequence typing (cgMLST) applicable to the monophyletic *Klebsiella oxytoca* species complex

**DOI:** 10.1128/jcm.01725-23

**Published:** 2024-05-23

**Authors:** Johanna Dabernig-Heinz, Gabriel E. Wagner, Karola Prior, Michaela Lipp, Sabine Kienesberger, Werner Ruppitsch, Torunn G. Rønning, Dag Harmsen, Ivo Steinmetz, Eva Leitner

**Affiliations:** 1 Diagnostic and Research Institute for Hygiene, Microbiology and Environmental Medicine, Medical University of Graz, Graz, Austria; 2 Department of Periodontology and Operative Dentistry, University Hospital Münster, Münster, Germany; 3 Institute of Molecular Biosciences, University of Graz, Graz, Austria; 4 BioTechMed-Graz, Graz, Austria; 5 Field of Excellence BioHealth, University of Graz, Graz, Austria; 6 Institute of Medical Microbiology and Hygiene, Austrian Agency for Health and Food Safety, Vienna, Austria; 7 Department of Medical Microbiology, St. Olavs Hospital, Trondheim University Hospital, Trondheim, Norway; 8 Department of Clinical and Molecular Medicine, Norwegian University of Science and Technology, Trondheim, Norway; Maine Medical Center Department of Medicine, Portland, Maine, USA

**Keywords:** cgMLST, *Klebsiella oxytoca*, nosocomial infections, species complex, resistance markers, virulence markers, molecular surveillance

## Abstract

The environmental bacterium *Klebsiella oxytoca* displays an alarming increase of antibiotic-resistant strains that frequently cause outbreaks in intensive care units. Due to its prevalence in the environment and opportunistic presence in humans, molecular surveillance (including resistance marker screening) and high-resolution cluster analysis are of high relevance. Furthermore, *K. oxytoca* previously described in studies is rather a species complex (KoSC) than a single species comprising at least six closely related species that are not easily differentiated by standard typing methods. To reach a discriminatory power high enough to identify and resolve clusters within these species, whole genome sequencing is necessary. The resolution is achievable with core genome multilocus sequence typing (cgMLST) extending typing of a few housekeeping genes to thousands of core genome genes. CgMLST is highly standardized and provides a nomenclature enabling cross laboratory reproducibility and data exchange for routine diagnostics. Here, we established a cgMLST scheme not only capable of resolving the KoSC species but also producing reliable and consistent results for published outbreaks. Our cgMLST scheme consists of 2,536 core genome and 2,693 accessory genome targets, with a percentage of good cgMLST targets of 98.31% in 880 KoSC genomes downloaded from the National Center for Biotechnology Information (NCBI). We also validated resistance markers against known resistance gene patterns and successfully linked genetic results to phenotypically confirmed toxic strains carrying the *til* gene cluster. In conclusion, our novel cgMLST enables highly reproducible typing of four different clinically relevant species of the KoSC and thus facilitates molecular surveillance and cluster investigations.

## INTRODUCTION

The opportunistic pathogen *Klebsiella oxytoca sensu lato* (s.l.) is a gram-negative bacterium of environmental origin that is associated with clinical infections in humans (1, 2). *K. oxytoca* s.l. is the second most common cause of clinical infections in the *Klebsiella* genus (1) after the notorious *Klebsiella pneumoniae* (2). Of particular concern is that both species show increasing rates of multidrug resistance and even share virulence factors (3). Particularly in healthcare-associated outbreaks, *K. oxytoca* poses a significant threat by nosocomial transmission of extended-spectrum-β-lactamase producing *K. oxytoca* in intensive care units (ICUs) (4) or infection of neonates in neonatal intensive care units (5). Such outbreaks and transmission events are particularly difficult to manage due to the persistence of the bacteria in and spread via environmental reservoirs (6) and opportunistic presence in humans (7, 8). The emergence of antibiotic-resistant strains in clusters makes molecular surveillance high priority to trace infectious agents, outbreak strains, and outbreak origins.

Traditional typing methods such as multilocus sequence typing (MLST) have limited discriminatory power that may not be suitable for elucidating outbreaks. However, high resolution of genetic differences is required to distinguish between both closely related isolates, indicating a common origin and thus potentially an outbreak, as well as between more distantly related isolates. Whole genome sequencing offers highest accuracy in the quantification of genetic distances by calling single nucleotide polymorphisms (SNPs) (9). This quantification serves as a basis for subsequent phylogenetic analyses of, e.g., evolutionary relationships or population dynamics. However, the lack of a consistent nomenclature complicates the exchange and comparison of results between laboratories. Furthermore, the more complex the analysis, the higher are the demands for bioinformatics knowledge and computational power. In the field of genomic pathogen surveillance, core genome multilocus sequence typing (cgMLST) has therefore established itself as an alternative. As a gene-by-gene approach, it relies on calling alleles of core genome genes instead of SNPs, which makes it more robust against recombination, but comes with a slightly lower discriminatory power compared to SNP approaches (10). The analysis is not limited to core genome genes and can easily be extended to include other targets of interest, such as resistance or virulence markers. The ability to create a shared database for comparability of results facilitates cross-laboratory and cross-national studies and initiatives (e.g., “PulseNet” or “Pathogenwatch”). Given its great potential in clinical microbiology, it is not surprising that the establishment of a variety of cgMLST schemes and their successful validation against other typing methods have been published recently (11–14). With advances in long-read sequencing for broad applicability (15), sequencing capacity is not limiting anymore. It can therefore be anticipated that cgMLST-based typing will be used even more extensively in the future, extending its application to further species, and increasing the need for further schemes. cgMLST analysis enhances MLST analysis by assigning allele numbers correlating to the exact genetic sequence of thousands of coding regions throughout the genome. The allelic profile for each strain is stored centrally and relationships between isolates can be assessed via standardized databases. The comparison of all genes in the core genomes provides a very high resolution, which enables global surveillance of circulating strains and also outbreak investigation. In addition to its discriminatory power and robustness, its user-friendly application, which requires little computational power or bioinformatics knowledge in contrast to SNP calling, makes cgMLST typing accessible on standard computers in many different settings.

The analysis of the *K. oxytoca* s.l. is complicated by the fact that it is rather a species complex (KoSC) than a single species that comprises several closely related species, including the clinically relevant *K. oxytoca*, *Klebsiella grimontii*, *Klebsiella michiganensis,* and *Klebsiella pasteurii* (3). Species differentiation is challenging due to similar clinical presentation and almost identical phenotypic characteristics of KoSC members (1) often leading to misidentification as *K. oxytoca* and possibly obscuring the clinical or epidemiological differences between these species.

Genotyping approaches using whole genome information like cgMLST also enable such a differentiation and assignment to the correct *Klebsiella* species by analysis of specific marker genes. The most commonly used gene for *K. oxytoca* s.l. species differentiation is the intrinsic β-lactamase gene *blaOXY* (16). The β-lactamase gene variants [*bla*_OXY-(1–9)_] are chromosomally encoded and have evolved separately for thousands of years (2, 17), making them a viable marker for differentiation within the KoSC (18).

KoSC members are well known for their ability to acquire resistance to multiple classes of antibiotics through resistance plasmid uptake (19) and acquisition of chromosomal mutations (16) demanding for a comprehensive monitoring. Next to the intrinsic β-lactamase gene (*bla*_OXY_), *K. pneumoniae* carbapenemase-2 gene (*bla*_KPC-2_), which confers resistance to carbapenems (16), is important to monitor as resistance genes promote the spread of carrying strains (7). Besides the distribution of antibiotic resistance markers (AMR), certain virulence factors that increase pathogenicity and fitness of KoSC strains are of interest (16). Toxigenic KoSC members, for example, encode capacity for biosynthesis of two enterotoxins tilimycin and tilivalline in the tilimycin/tilivalline (*til*) gene cluster (20), alternatively called kleboxymycin biosynthesis gene cluster (21). Cytotoxic KoSC members are directly linked to antibiotic-associated hemorrhagic colitis (AAHC) (22–24) and are associated with necrotizing enterocolitis (NEC) in premature infants (25), raising interest in prevalence and distribution of the *til* genes among clinical isolates.

To combat the spread of antibiotic-resistant *Klebsiella* sp. strains, it is crucial to identify outbreak strains and potential resistance markers and monitor the spread of these strains and genes. Despite the significant medical demand for fast and user-friendly molecular surveillance of KoSC and related outbreaks, there is no public cgMLST scheme available for neither *K. oxytoca* nor the complete KoSC yet. Here, we established a novel cgMLST scheme for the KoSC, which enables species differentiation, genotyping of AMR and virulence factors, and strain resolution with high discriminatory power. Moreover, we provide a standardized and central nomenclature of alleles for easy application and data exchange.

## MATERIALS AND METHODS

### cgMLST target scheme definition

A two-step process was used to account for the overall variability of the KoSC. First, the concatenated sequences of all available *K. oxytoca* s.l. sequencing types (STs) (*n* = 421, as of 4 November 2022) were downloaded from the MLST website (https://pubmlst.org/organisms/klebsiella-oxytoca) (26) and used for a Bayesian analysis of population structure (BAPS) (27) as previously described (11–13) with one minor modification. ST105 was excluded from the analysis, as it had a great phylogenetic distance to all other sequence types in the maximum-likelihood tree and was revealed as an incorrectly deposited *Raoultella* sp. isolate.

Next, all genome data sets available for monophyletic KoSC members at NCBI (*K. oxytoca, n* = 300; *K. michiganensis, n* = 410; *K. grimontii, n* = 155; *K. pasteurii, n* = 15, as of 4 November 2022) were downloaded, analyzed with the SeqSphere+ v.8.99.1 [Ridom GmbH (28)] MLST task template, and assigned to their corresponding BAPS partitions if an ST could be extracted from the data. In addition, a fastANI (Wingett) analysis (29) was performed using the NCBI reference strain of the respective species (*K. oxytoca,* NZ_CP033844.1; *K. michiganensis,* NZ_AP022547.1; *K. grimontii,* NZ_LR607336.1; *K. pasteurii,* NZ_CP089403.1; as of 4 November 2022) for comparison. For every BAPS partition, the data set with the highest NCBI genome status (in the order “complete,” “chromosome,” “scaffold,” “contig”) and the highest fastANI value was selected as a query genome to build a preliminary scheme. For that, the cgMLST Target Definer v.1.5 (windows with default settings) function implemented in the SeqSphere+ software was used with the NCBI reference strain *K. oxytoca* FDAARGOS_500 (NZ_CP033844.1) as seed genome (Table S1).

Available plasmid sequences of *K. oxytoca* (*n* = 46), *K. michiganensis* (*n* = 36), *K. grimontii* (*n* = 19), and *K. pasteurii* (*n* = 1) were excluded in the target definer settings (as of 4 November 2022).

In a second step, the scheme was optimized to enable the applicability to the four different species. For this, all NCBI records were reanalyzed using the preliminary cgMLST scheme. All targets identified in less than 95% of the analyzed data sets were transferred to the Accessory Task Template to ultimately retain the finalized cgMLST.

### KoSC strains and genomes

The KoSC cgMLST scheme was validated using in total 65 strains, comprising 33 newly sequenced isolates and 32 obtained from NCBI or colleagues, as listed in detail in Table S2. The isolates were part of three studies discussing recent outbreaks: 28 isolates from Norway (30) including seven control and one reference strain NZ_AP014951, seven isolates from Graz in 2010 (4), including two control strains, and 14 isolates from Graz in 2013 (7), including three control strains. In addition, we sequenced five toxigenic and seven non-toxigenic strains from the Graz strain collection. Toxicity of these strains was assessed by MTT assays as previously described (23). These strains cover a variety of different STs belonging to different BAPS partitions and also one new ST without BAPS partition assignment. Moreover, two *K. pasteurii* (Sb-24 and Kox205) and two *K. grimontii* (CP055412 and CP044527) were included in the analysis to validate the assignment of rarer KoSC species.

### DNA isolation, whole genome sequencing, and assembly

DNA isolation was performed with the Blood and Tissue kit (Qiagen). Purity of the genomic DNA was checked with the Nano Drop 2000 device (Thermo Scientific) and concentration of the isolated genomic DNA was determined with the Qubit 2.0 instrument using the Qubit dsDNA BR Assay (Life Technologies, Invitrogen division). Libraries for whole genome sequencing were prepared using the Nextera XT chemistry (Illumina, Inc.) for a 300 bp paired-end sequencing run on an Illumina MiSeq sequencer following Illumina’s recommended standard protocols. Illumina raw data were *de novo* assembled in SeqSphere+ (version 7.8.0, Ridom GmbH) (28) with the implemented SKESA assembler version 2.3.0 (31) with default settings.

### cgMLST scheme validation and evaluation

KoSC isolates were typed with our newly established cgMLST scheme in SeqSphere+. Allelic profiles were generated by assigning allele numbers based on the genetic sequence of the respective loci. Also, all 65 isolates were assessed for the β-lactamase and carbapenemase resistance genes *blaOXY*, *blaKPC,* and *blaTEM* in SeqSphere+ with curated allele libraries from the Institute Pasteur [https://bigsdb.pasteur.fr/cgi-bin/bigsdb/bigsdb.pl?db=pubmlst_klebsiella_seqdef&page=alleleQuery&locus=blaOXY*,* accessed on 5 March 2023 (26)]. Another query created in SeqSphere+ consists of 12 genes relevant to the functionality of the *til* gene cluster based on the toxin-producing reference strain *K. oxytoca* AHC-6 (CP098757) (32). Results from this genetic analysis were evaluated with results obtained from *in vitro* toxicity assays. The cluster threshold, which is a simple estimate of whether to include an isolate in an outbreak or not, was determined by evaluating minimum spanning trees (MSTs) of the three confirmed outbreaks.

Our MLST results of *phoE* had to be reconfirmed by Sanger sequencing due to ambiguities with former published alleles of the Graz outbreak strains. The difference between the former results and our cgMLST in those alleles was based on a mutation within the primer binding site of the original primer that was overlapping the analyzed region of *phoE*.

### SNP calling

Snippy (version 4.6.0) was used to perform SNP calling using the raw reads and the same reference as used in the cgMLST typing scheme. The core SNPs were identified with snippy-core and recombinant regions were identified and masked with gubbins (version 3.3.1). The generated trees were visualized with Phandango (33).

### Phylogenetic trees

MSTs and neighbor-joining trees were built with SeqSphere+ based on the comparison of allele variants for the respective strain. Distance between strains is based on the number of different alleles present. An MST represents a minimal distance of all strains in the tree and facilitates the investigation of closely related outbreak strains.

## RESULTS AND DISCUSSION

### cgMLST target scheme definition

The cgMLST for the KoSC members was built with additional steps to meet the high-resolution requirements within the highly variable species complex. Variability of the analyzed complex members was assessed by a BAPS analysis of 405 STs downloaded from the MLST website (26). Analysis revealed seven partitions and exhibited that eight of the 405 STs had significant admixture, as they could only be assigned to one of these partitions with a probability of less than 95%. So finally, 397 STs were allocated to one of seven BAPS partitions. Subsequently, we analyzed the STs in 880 genomic data sets downloaded from NCBI with SeqSphere+ and were able to assign 664 isolates to one of the BAPS partitions based on their ST. The isolate with the best data set, comprising the highest genome status as well as the highest fastANI score, of each BAPS partition was manually selected as query genome for the preliminary cgMLST target scheme. For one BAPS partition, no isolate with a matching ST could be included as no hit was found within the 880 analyzed NCBI data sets. Seed and query genome data sets used for defining the preliminary cgMLST target scheme comprising 2,727 targets and covering 47% of the seed genome are listed in Table S1.

During the refinement step, in which the entire 880 NCBI genome data sets were analyzed using the preliminary cgMLST scheme, an additional 191 targets were manually moved from the core to the accessory genome task template because they were present in less than 95% of the data sets. Our final cgMLST scheme thus comprised 2,536 targets covering 43% of the seed genome, while the accessory template consisted of a total of 2,693 targets. Reanalysis of the 880 data sets of four different species with the final cgMLST scheme resulted in a mean percentage of good targets of 98.31% across the KoSC species. Such a high typeability is generally a good indicator for the applicability of the cgMLST scheme and the representativeness of the scheme for different strains and even for different species of the KoSC. At least two additional species have been proposed for the KoSC, *Klebsiella huaxiensis* and *Klebsiella spallanzani*, but they have minimal representation in databases due to not being associated with human infection, complicating accurate conclusions about their typeability with our scheme (3, 17).

### Molecular surveillance using the KoSC cgMLST scheme

Detailed typing results of the 65 assemblies are listed in Table S2. The percentage of good targets of the cgMLST scheme in the isolates of four different species is on average 99.5%, which equals 12 missing targets in core genome on average.

As expected, missing values vary in a species-specific manner in the accessory targets. Due to genes exclusive or clustered in the respective species, *K. oxytoca* have a median 363 ± 34.5 missing values in the accessory task template, *K. michiganensis* 1,728 ± 66.5, *K. grimontii* 1,934 ± 17.8, and *K. pasteurii* 2,041 ± 0.82. Our data set spans 20 diverse sequencing types. For three of the 65 analyzed strains, we could not assign an ST, once because there was a new ST allele combination that was newly deposited, and the rest because of missing alleles for some MLST targets.

### Validation of cgMLST scheme against other methods with published outbreak event

The first evaluation with confirmed outbreak strains was based on a nosocomial outbreak caused by antibiotic-susceptible *Klebsiella oxytoca* in an intensive care unit in Norway in 2016 (30). The former results from an allele-specific PCR assay for *infB*-identifying outbreak isolates were confirmed by our analysis. As expected, the locus of *infB* of our cgMLST scheme yielded the exact same allele for all 20 isolates of the outbreak strain but not for the seven control isolates (Table S2). Additionally, the neighbor-joining tree of isolates in our high-resolution analysis based on all cgMLST targets showed the same pattern as the nucleotide core alignment in the former study (see Fig. S1), reconfirming the applicability of the here established cgMLST scheme. No resistance genes were found, which is consistent with the published antibiotic susceptibility phenotype of the isolates (30).

**Fig 1:**
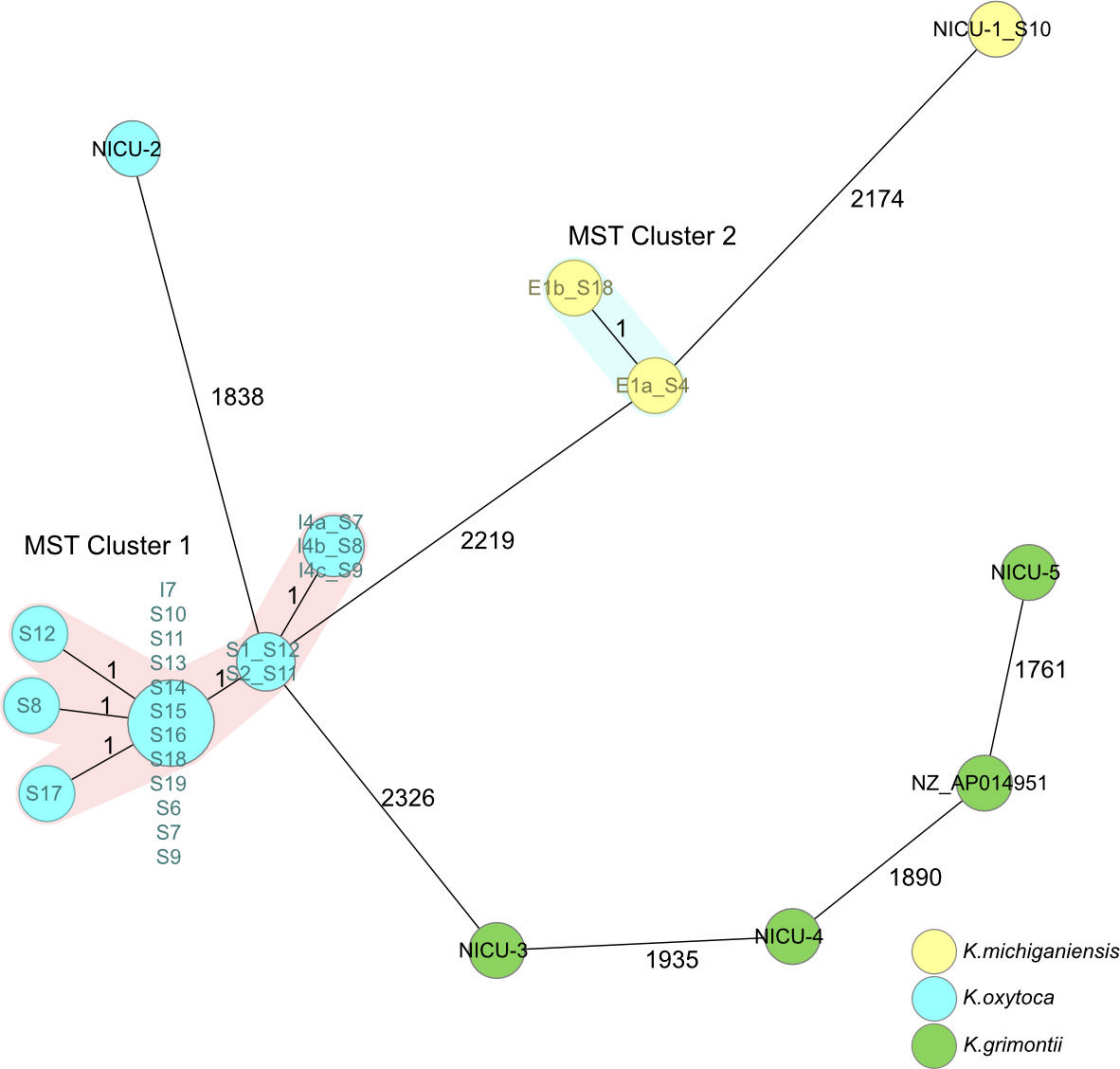
MST of 28 isolates from a nosocomial outbreak investigation (30). The distance is based on 2,536 target genes with pairwise ignore missing values. Isolates are colored according to the species. Clustering of outbreak strains is visible (MST cluster 1). Also, two environmental isolates from the same location cluster together (cluster 2) and the remaining environmental and reference strains of this study are distant to all other strains, but still cluster on species level (as indicated by the color of nodes).

In Fig. 1, we visualized the outbreak isolates in a minimum spanning tree, which shows the maximum genetic distance of one differing allele between outbreak isolates in MST cluster 1. Also, environmental and patient isolates belonging to different species of the KoSC are located closest to isolates of the same species in the MST, which indicates discrimination of the KoSC with the cgMLST. The maximum genetic distance between outbreak isolates is three alleles based on a distance matrix between all strains. All other isolates have minimal distances of about 2,000 different loci between them in this specific outbreak study, except for a replicate from the same environmental source (MST cluster 2 in Fig. 1).

### cgMLST improves the resolution to resolve outbreak strains

The next validation of our established KoSC cgMLST scheme was carried out on two related nosocomial outbreaks of multi-resistant *Klebsiella oxytoca* in Graz (4, 7). From the first study, we analyzed five isolates from ICU patients as well as two wild-type susceptible control isolates from 2003 and 2011, both from the same hospital, but unrelated to the outbreak, for a more sensitive comparison. The outbreak isolates were all *Klebsiella pneumoniae* carbapenemase (KPC)-producing strains and indistinguishable according to automated repetitive PCR with a DiversiLab instrument in the original study [Fig. 1 in publication (4)]. Our next-generation sequencing (NGS) analysis confirmed KPC production via allele assignment and comparison with the Institute Pasteur allele libraries and AMR gene finder implemented in SeqSphere+, which yielded *blaKPC*-2 (allele 2) in all outbreak isolates but not for control isolates. A neighbor-joining tree (Fig. S2) of the seven isolates is in agreement with the previous study showing a dedicated cluster of outbreak strains and with both control isolates as clearly separate outgroups. The reported similarity between isolates was 99.4%–99.6% with DiversiLab, which is similar to SeqSphere+’s similarity which was between 99.84 and 99.96% of matching loci between outbreak strains.

From the subsequent outbreak on a hematology ward (7), we investigated 11 outbreak isolates, consisting of seven patient and four environmental samples. Additionally, three challenging control isolates derived from different samples (Bronchoalveolar lavage, blood, and stool) with a more susceptible antibiotic resistance phenotype but from the same time period and hospital as the outbreak isolates were chosen as controls. One of those isolates even had the same ST as the outbreak isolates, but nevertheless did not belong to the outbreak epidemiologically. In line with former MLST results, our analysis in SeqSphere+ also yielded the same ST for all of the outbreak isolates, namely ST199, which is the updated ST for the Graz strains based on our NGS data and validated with Sanger sequencing (for details, see Materials and Methods).

**Fig 2:**
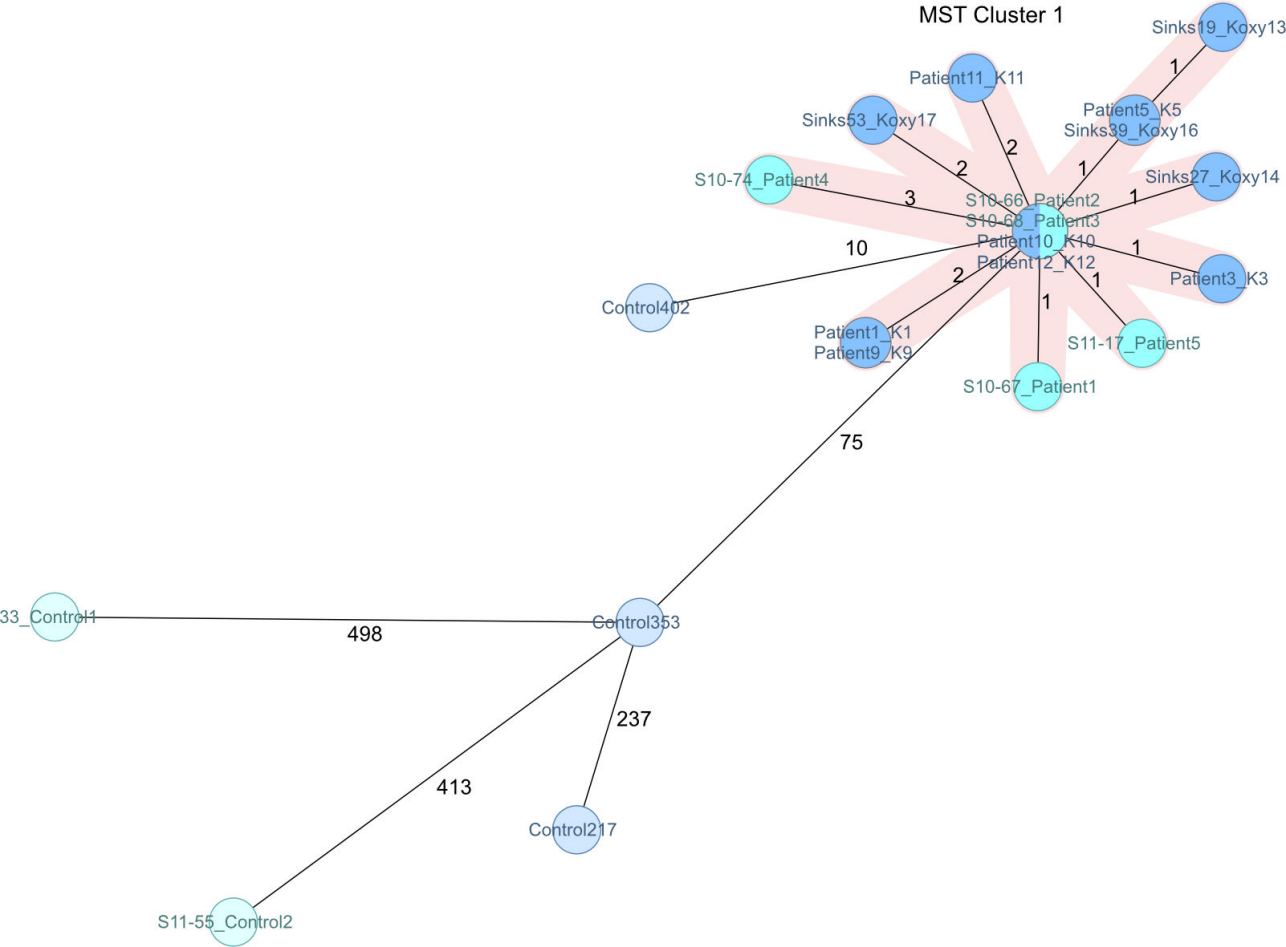
Minimum spanning tree of 21 isolates from a neonatal intensive care unit in Graz from 2010 to 2013 (4, 7), and controls based on 2,536 target genes with pairwise ignore missing values. The close relatedness of all outbreak strains in MST cluster 1 is visible. Strains mentioned in the first outbreak investigation are colored cyan, isolates from the second one are colored blue. Control strains from either investigation are marked with the respective faded color.

As all isolates from both published studies in Graz (4, 7) belong to the same outbreak, we included all isolates in a single comprehensive minimum spanning tree. All isolates from the first outbreak in 2010 (4) and the subsequent in 2013 (7) form a single cluster, containing all patient and sink samples identified as a reservoir (Fig. 2). All controls are clearly separated from the outbreak cluster despite the challenging similarities, which is furthermore in line with the results of our SNP analysis (see Fig. S3 and S4). Of note, strains belonging to the cluster were deemed indistinguishable by the former studies (4, 7) and are now mostly resolved, demonstrating the higher resolution of our typing approach. However, to increase discriminatory power, users might resort to SNP analyses or whole genome MLST, which can further resolve isolates.

One isolate epidemiologically uncorrelated to the outbreak but from the same hospital, period, and ST was deliberately chosen as a hard control (Control 402). Indeed, it has less allelic differences to the cluster than any other control in our whole study, with a minimal distance of only 10 allelic differences to the closest outbreak isolate. The analysis of the allelic distance between all outbreak isolates and the controls enables the establishment of a cluster threshold that is chosen to include all outbreak isolates in a cluster based on this distance, but excludes genetically close controls like the mentioned control isolate. Here, we propose a flexible threshold between six and nine allelic differences, as the maximum difference between outbreak isolates is six, considering every possible pairwise comparison and the closest distance between a control and an outbreak isolate is 10. Which exact threshold is applied to a data set depends on the scope of the study. Thus, a more conservative threshold of six reduces the likelihood of false inclusion of non-outbreak strains, hence increasing specificity, and conversely, the upper limit of nine can be used for higher sensitivity. It is important to emphasize that the cluster threshold is a soft criterion that enables a simple initial classification. Epidemiological data must always be taken into account to assess whether a strain belongs to an outbreak or not.

### *K. oxytoca* species complex differentiation

Species assignment via the implemented and automated marker screening (*blaOXY* gene) agreed with NCBI species designation for analyzed genomes. According to the *blaOXY* alleles, the 12 isolates from different BAPS partitions included the following species: three *K*. *oxytoca* (*bla*_OXY-2_), two *K*. *grimontii* (*bla*_OXY-6_), six *K*. *michiganensis* (*bla*_OXY-1_ and *bla*_OXY-5_), and one *K*. *pasteurii* (*bla*_OXY-4_). These results were compared to the top species match identity by “Mash Screen” (34), which might serve as a first overlook of *K. oxytoca* (*n* = 4), *K. grimontii* (*n* = 1), and *K. michiganensis* (*n* = 7), but is not ideally suited for species differentiation within the KoSC as it leads to incorrect assignments of four strains, one of them on genus level as *Escherichia coli*. All isolates from the outbreaks we investigated (4, 7, 30) and the control isolates from Graz were reconfirmed as *K. oxytoca* (*bla*_OXY-2_). The non-outbreak isolates from Norway, serving as controls, included one *K. oxytoca* (*bla*_OXY-2_), three *K. michiganensis* (*bla*_OXY-1_), and three *K. grimontii* (*bla*_OXY-6_), which were again mostly misidentified as *K. oxytoca* by “Mash Screen,” as seen in Table S2.

### Plasmids, virulence factors, and resistance markers

A newly implemented feature of SeqSphere+ allows the automated and thorough analysis of plasmids and their resistance genes. As the assemblies were highly fragmented (mean 139 fragments ± 112), plasmid prediction was difficult and should be considered with caution. The prediction varied greatly between strains (0–11 plasmids, median 2 ± 2.3). Nevertheless, a first insight of plasmid-associated resistances could be obtained by the Plasmid AMRFinderPlus results in Table S3. Despite the fragmentation, the antimicrobial resistance gene *blaKPC*-2 was identified on plasmids in all of the Graz outbreak isolates, posing a risk of transmission. The *blaKPC*-2 gene was absent from all control isolates from Graz and every isolate from the Norwegian study. The absence of resistance in a closely related isolate with the same ST in Graz substantiates this suspicion and indeed points to acquired carbapenem resistance in the isolates of the Graz outbreak. KoSC strains are in general intrinsically resistant to β-lactams. In addition to the blaOXY variants, genetic analysis revealed several AMR markers including *oqxA*/*oqxB or blaOXA*/*blaTEM*-1, notably present in multi-resistant strains from the Graz outbreaks (4, 7). Thus, results from genetic analysis of the resistance genes reconfirm multi-resistance and possible extended-spectrum β-lactamase activity that is however phenotypically superimposed by KPC-2 activity. All outbreak isolates and one control isolate from Graz carried 11–12 genes associated with antibiotic resistance, while the remaining isolates from Graz and Norway carried three to four such genes. A table of all resistance genes identified using the implemented AMR finder in SeqSphere+ is attached as Table S3.

The presence of the *til* gene cluster is a valuable genetic marker, since the toxicity of strains has been linked to AAHC in previous studies (22, 23) and has been associated with NEC (5, 25). Presence of *til* genes was compared to strain toxicity as evaluated by MTT assays. All of the phenotypically toxin-negative strains (*n* = 7) lack the 12 genes necessary for toxin production. In contrast, for all toxigenic isolates (*n* = 5), we confirmed the presence of either all 12 but at least 10 of the *til* genes (Table S4). The genes missing in the toxigenic isolates were either *npsC*, which is too small (393 bp) of reliable detection, and/or *hmoX*, known to be non-essential for toxin production (20).

In summary, analysis of all 65 isolates suggests the presence of an active *til* cluster in 52.3% of the strains (34/65) (Table S4). Among the 34 positive isolates, 22 scored positive for all relevant genes and 12 isolates were only missing *hmoX* and/or *npsC*. All isolates analyzed from the Graz outbreaks carried no *til* gene cluster. In contrast, all outbreak isolates from Norway were genetically positive for toxin production, indicating a possibly elevated enterotoxicity of these isolates (30). Three genetically *til* gene negative (all *K.michiganensis*) and five *til* gene positive isolates were identified among the control strains from Norway. In the literature, a similar picture of presence of the *til* gene cluster in the KoSC is drawn with different percentages based on the specific species (less frequent in *K. michiganensis*) and, in general, a presence in about 50% of all KoSC isolates is analyzed per study (1, 17).

### Conclusion

Here, we present a newly implemented and established cgMLST scheme for the *Klebsiella oxytoca* species complex. Through our extensive evaluation, we have demonstrated the versatility of our cgMLST scheme for the genetic characterization of four clinically relevant species of the KoSC ranging from nosocomial outbreak investigations of multidrug-resistant strains to AMR gene and tilivalline cluster identification. The validation with isolates from previously published outbreak studies utilizing other typing methods such as DiversiLab or pulsed-field gel electrophoresis clearly demonstrates the higher resolution of our cgMLST scheme for the identification of genetic differences between closely related strains and the characterization of specific target genes. This enhanced resolution allows for more precise cluster investigations, thus contributing to the ability to classify and analyze outbreaks of KoSC strains. We propose a cluster threshold of six to offer a guideline and facilitate cluster investigation in future studies. By providing standardized and reproducible results across different laboratories and studies, our final cgMLST scheme offers a quick response to KoSC-related public health concerns. The user-friendly application and availability of open source software for typing (e.g., Chewbacca or pyMLST) further improve accessibility and usability and makes the cgMLST scheme established here a valuable contribution to the genomic surveillance of KoSC isolates.

## Data Availability

Newly sequenced raw read data has been deposited under BioProject No. PRJNA1055729 in the National Center for Biotechnology Information Sequence Read Archive repository. Corresponding accession numbers for each isolate are included in Table S2.

## SUPPLEMENTAL MATERIAL

Supplemental material jcm.01725-23-s0001.pdf Table S1; Fig. S1 to S4.

Table S2 jcm.01725-23-s0002.pdf All isolates examined in our study, with metadata like association, species, and MLST results.

Table S3 jcm.01725-23-s0003.pdf All isolates and their respective results regarding antibiotic resistance and plasmids.

Table S4 jcm.01725-23-s0004.pdf All isolates and their respective results regarding the til gene cluster.

ASM does not own the copyrights to Supplemental Material that may be linked to, or accessed through, an article. The authors have granted ASM a non-exclusive, world-wide license to publish the Supplemental Material files. Please contact the corresponding author directly for reuse.
