# Supplementary material for: Core genome multilocus sequence typing (cgMLST) applicable to the monophyletic Klebsiella oxytoca species complex
Source: J Clin Microbiol. 2024 May 23;62(6):e01725-23. doi: 10.1128/jcm.01725-23 (PMC11237601; doi:10.1128/jcm.01725-23)
Supplement: Supplemental material — Table S1; Fig. S1 to S4. [file jcm.01725-23-s0001.pdf]

Supplementary

Methods

Supplementary Table 1: NCBI datasets used as Seed and Penetration query genomes for the generation of the preliminary cgMLST scheme for the K. oxytoca complex with the SeqSphere+ Target Definer. No isolates with MLST STs included in BAPS partition 1 (ST272, 273, 274, 276) were available in pubMLST database, also there was no hit within analyzed NCBI datasets (n=880). FastANI similarity is calculated with the respective reference of the species as mentioned in the methods.

BAPS partition	Strain	Species	FastANI similarity (%) to reference	MLST ST	Genome Status	Genome Accession
BAPS2	FDAARGOS_500*	*K. oxytoca*	100,00	59	CompleteGenome	NZ_CP033844.1
BAPS3	M5a1	*K. grimontii*	99,38	104	CompleteGenome	NZ_CP063301.1
BAPS4	Kox205	*K. pasteurii*	100,00	300	CompleteGenome	NZ_CP089403.1
BAPS5	5088	*K. michiganensis*	98,69	11	CompleteGenome	NZ_CP103554.1
BAPS6	F107	*K. michiganensis*	98,06	383	CompleteGenome	NZ_CP024643.1
BAPS7	BSI-KPN166	*K. michiganensis*	99,05	40	CompleteGenome	CP102103.1

* strain used as seed

Results

The neighbour joining tree (Supplementary Figure 1, compare to Figure 2 in Rønning *et* al. (30)) confirms NICU isolates from faecal specimens to be different from the outbreak strain, as well as the similarity of the genotype of two environmental isolates from one sink.

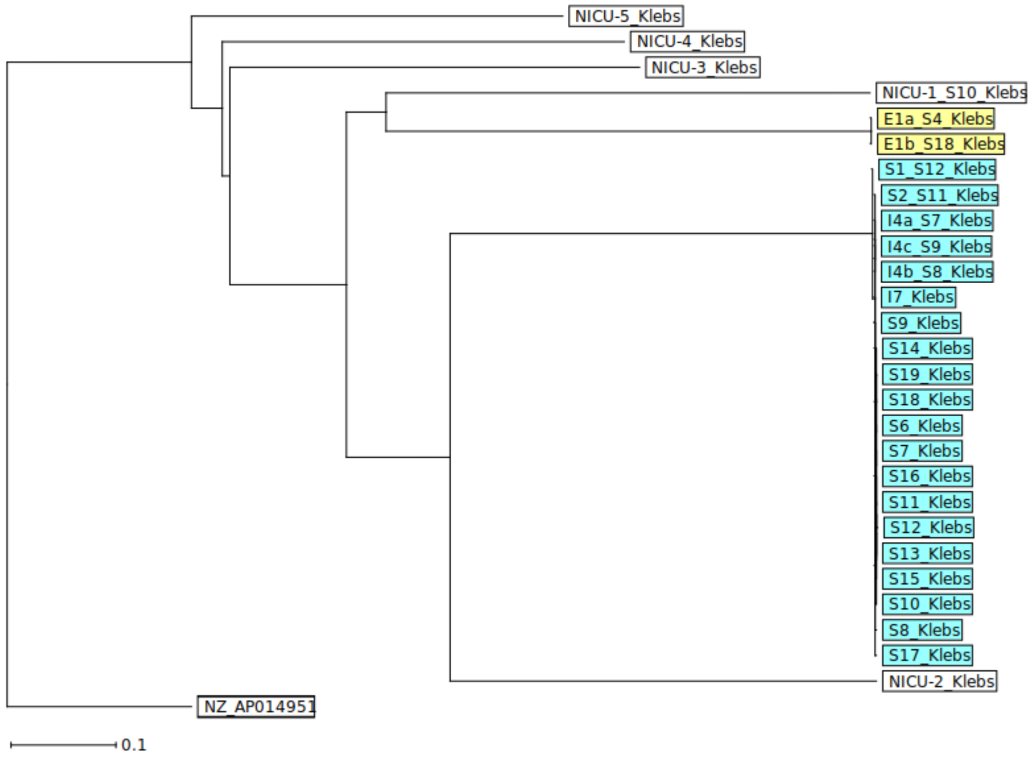

Supplementary Figure 1: Neighbour joining tree of 28 isolates from Norway and the reference strain used in their analyses (30). Yellow marked isolates are closely related environmental strains, cyan marked are outbreak isolates and unmarked isolates are control isolates.

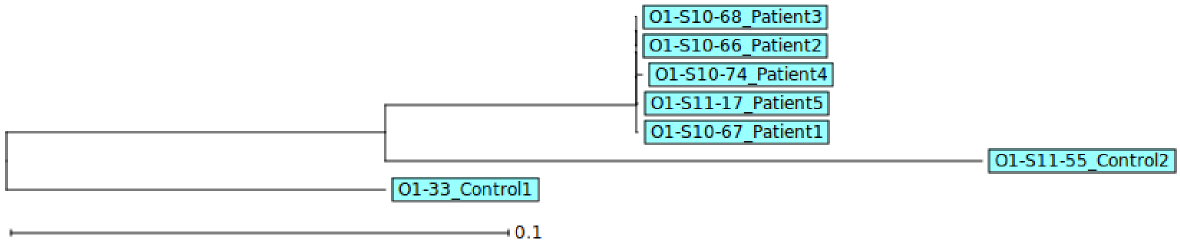

Supplementary Figure 2: Neighbour joining tree of seven Graz isolates from 2010 (4) including the two control isolates. Patient isolates form a cluster separated from the two controls.

For further evaluation of our cgMLST scheme, we performed a SNP analysis of the Graz outbreak. As shown in the trees in Supplementary Figures 3 and 4, both methods allow the identification of the outbreak isolate cluster as a single branch (highlighted in blue) separated from the controls. This reconfirms the suitability of the selected cgMLST approach for bacterial typing purposes. Nevertheless, if necessary, SNP analyses still offer the highest discriminatory power, as expected.

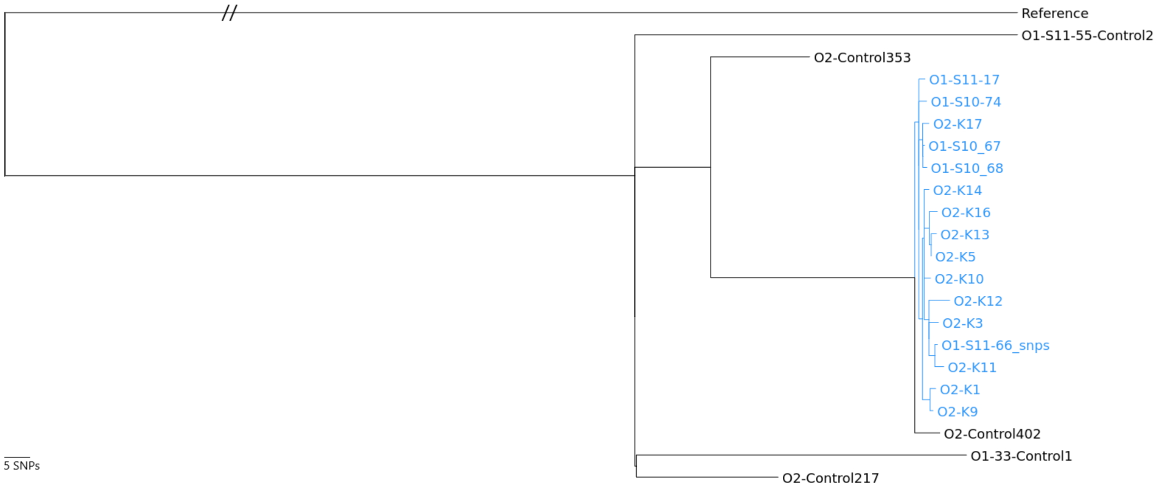

Supplementary Figure 3: SNP-based neighbour joining tree of all Graz outbreak isolates shows the outbreak cluster as a single branch highlighted in blue that is separated from the controls. This tree is rooted to the same reference (NZ_CP033844_1) as the cgMLST-based tree below, but the distance to the reference is not up to scale for visualisation purposes.

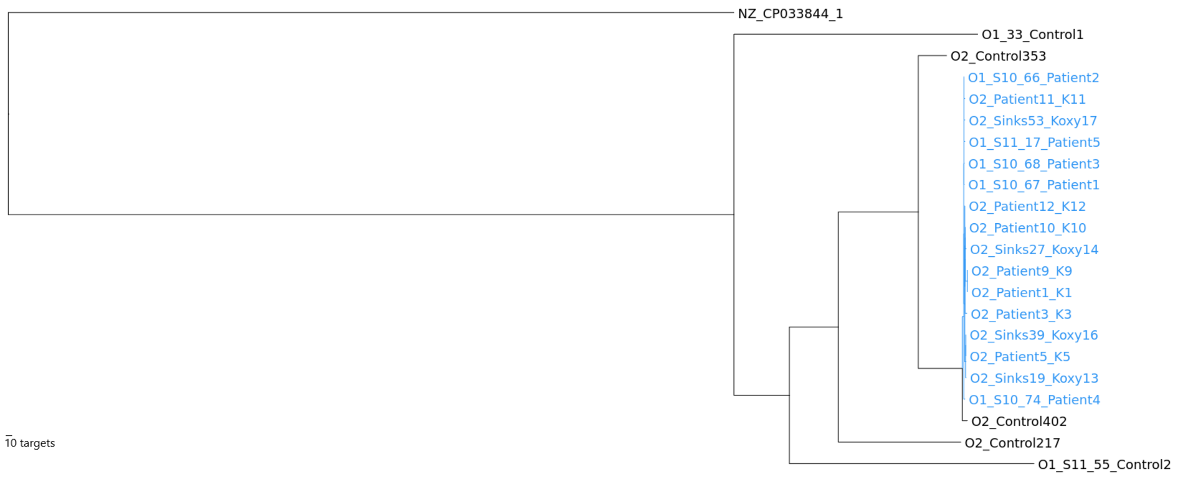

Supplementary Figure 4: cgMLST-based neighbour joining tree of all Graz outbreak isolates shows the outbreak cluster as a single branch highlighted in blue that is separated from the controls. The tree is rooted to NZ_CP033844_1.
