# Supplementary material for: Core genome multilocus sequence typing (cgMLST) applicable to the monophyletic Klebsiella oxytoca species complex
Source: J Clin Microbiol. 2024 May 23;62(6):e01725-23. doi: 10.1128/jcm.01725-23 (PMC11237601; doi:10.1128/jcm.01725-23)
Supplement: Table S2 — All isolates examined in our study, with metadata like association, species, and MLST results. [file jcm.01725-23-s0002.pdf]

Supplementary Table 2: A list of all examined isolates included in our study with their respective metadata like association to the different outbreaks and accession numbers. Also included are various cgMLST results like species assignment via the blaOXY gene assignment (as mentioned in the results) and mash screen, missing values in the cgMLST and the percentage of good targets of all the core genome targets. Also the sequence type and the alleles of the seven loci it consists of are listed.

No.	Sample ID	Association (Reference)	Relation to Outbreak	Accession	acquired	blaOXY	Species (bla Oxy)	Top Species Match	#Missing values Core	#Missing values Accessory	Perc. Good Targets	Avg. Coverage (Assen	ST	gapA	infB	mdh	pgi	phoE	rpoB	tonB
1	#306_neg	Graz strain collection	-	SAMN39061525	newly sequenced	blaOXY_1-1	K. michiganensis	K. michiganensis	7	1696	99.7	145	27	3	5	7	13	17	4	5
2	#75_neg	Graz strain collection	-	SAMN39061526	newly sequenced	blaOXY_1-1	K. michiganensis	K. michiganensis	1	1675	100	162	11	3	5	7	8	11	6	5
3	15_neg	Graz strain collection	-	SAMN39061527	newly sequenced	blaOXY_5-2	K. michiganensis	K. michiganensis	60	1870	97.6	126	82	14	24	15	8	18	6	4
4	25_pos	Graz strain collection	-	SAMN39061528	newly sequenced	blaOXY_6-1	K. grimontii	K. oxytoca	5	1935	99.8	146	377	4	6	19	10	46	8	31
5	#119_pos	Graz strain collection	-	SAMN39061529	newly sequenced	blaOXY_1-20	K. michiganensis	K. michiganensis	3	1692	99.9	155	35	3	8	17	7	20	6	13
6	#21_neg	Graz strain collection	-	SAMN39061530	newly sequenced	blaOXY_1-9	K. michiganensis	K. michiganensis	10	1740	99.6	140	40	3	8	20	7	23	14	18
7	#19_neg	Graz strain collection	-	SAMN39061531	newly sequenced	blaOXY_2-21	K. oxytoca	K. oxytoca	7	375	99.7	163	78	1	22	2	27	9	1	2
8	#311_pos	Graz strain collection	-	SAMN39061532	newly sequenced	blaOXY_6-2	K. grimontii	K. grimontii	7	1967	99.7	157	216	4	6	19	10	46	24	31
9	#335_pos	Graz strain collection	-	SAMN39061533	newly sequenced	blaOXY_4-1	K. pasteurii	K. michiganensis	12	2040	99.5	146	new	17	23	23	39	69	30	43
10	21_neg	Graz strain collection	-	SAMN39061534	newly sequenced	blaOXY_5-5	K. michiganensis	K. michiganensis	8	1832	99.7	176	80	3	4	15	8	12	3	4
11	#118_pos	Graz strain collection	-	SAMN39061535	newly sequenced	blaOXY_2-1	K. oxytoca	K. oxytoca	2	325	99.9	154	19	1	2	2	1	16	1	2
12	#218_neg	Graz strain collection	-	SAMN39061536	newly sequenced	blaOXY_2-21	K. oxytoca	K. oxytoca	5	398	99.8	144	58	1	2	2	1	4	1	2
13	S10_Klebs	Norway Outbreak (30)	Outbreak Strain	Rønning *et* al. (30)	provided as fastq	blaOXY_2-2	K. oxytoca	K. oxytoca	2	347	99.9	113	179	1	36	2	1	66	2	2
14	S11_Klebs	Norway Outbreak (30)	Outbreak Strain	Rønning *et* al. (30)	provided as fastq	blaOXY_2-2	K. oxytoca	K. oxytoca	1	347	100	120	179	1	36	2	1	66	2	2
15	S12_Klebs	Norway Outbreak (30)	Outbreak Strain	Rønning *et* al. (30)	provided as fastq	blaOXY_2-2	K. oxytoca	Escherichia coli	39	353	98.5	60	179	1	36	2	1	66	2	2
16	S13_Klebs	Norway Outbreak (30)	Outbreak Strain	Rønning *et* al. (30)	provided as fastq	blaOXY_2-2	K. oxytoca	K. oxytoca	1	346	100	118	179	1	36	2	1	66	2	2
17	S14_Klebs	Norway Outbreak (30)	Outbreak Strain	Rønning *et* al. (30)	provided as fastq	blaOXY_2-2	K. oxytoca	K. oxytoca	2	348	99.9	108	179	1	36	2	1	66	2	2
18	S15_Klebs	Norway Outbreak (30)	Outbreak Strain	Rønning *et* al. (30)	provided as fastq	blaOXY_2-2	K. oxytoca	K. oxytoca	1	348	100	129	179	1	36	2	1	66	2	2
19	S16_Klebs	Norway Outbreak (30)	Outbreak Strain	Rønning *et* al. (30)	provided as fastq	blaOXY_2-2	K. oxytoca	K. oxytoca	1	346	100	103	179	1	36	2	1	66	2	2
20	S17_Klebs	Norway Outbreak (30)	Outbreak Strain	Rønning *et* al. (30)	provided as fastq	blaOXY_2-2	K. oxytoca	K. oxytoca	2	349	99.9	97	179	1	36	2	1	66	2	2
21	S18_Klebs	Norway Outbreak (30)	Outbreak Strain	Rønning *et* al. (30)	provided as fastq	blaOXY_2-2	K. oxytoca	K. oxytoca	3	349	99.9	169	179	1	36	2	1	66	2	2
22	S19_Klebs	Norway Outbreak (30)	Outbreak Strain	Rønning *et* al. (30)	provided as fastq	blaOXY_2-2	K. oxytoca	K. oxytoca	3	349	99.9	168	179	1	36	2	1	66	2	2
23	S1_S12_Klebs	Norway Outbreak (30)	Outbreak Strain	Rønning *et* al. (30)	provided as fastq	blaOXY_2-2	K. oxytoca	K. oxytoca	182	567	92.8	24	179	1	36	2	1	66	2	2
24	S2_S11_Klebs	Norway Outbreak (30)	Outbreak Strain	Rønning *et* al. (30)	provided as fastq	blaOXY_2-2	K. oxytoca	K. oxytoca	12	363	99.5	93	179	1	36	2	1	66	2	2
25	S6_Klebs	Norway Outbreak (30)	Outbreak Strain	Rønning *et* al. (30)	provided as fastq	blaOXY_2-2	K. oxytoca	K. oxytoca	2	348	99.9	121	179	1	36	2	1	66	2	2
26	S7_Klebs	Norway Outbreak (30)	Outbreak Strain	Rønning *et* al. (30)	provided as fastq	blaOXY_2-2	K. oxytoca	K. oxytoca	2	347	99.9	129	179	1	36	2	1	66	2	2
27	S8_Klebs	Norway Outbreak (30)	Outbreak Strain	Rønning *et* al. (30)	provided as fastq	blaOXY_2-2	K. oxytoca	K. oxytoca	2	348	99.9	154	179	1	36	2	1	66	2	2
28	S9_Klebs	Norway Outbreak (30)	Outbreak Strain	Rønning *et* al. (30)	provided as fastq	blaOXY_2-2	K. oxytoca	K. oxytoca	2	347	99.9	118	179	1	36	2	1	66	2	2
29	I4a_S7_Klebs	Norway Outbreak (30)	Outbreak Strain	Rønning *et* al. (30)	provided as fastq	blaOXY_2-2	K. oxytoca	K. oxytoca	4	352	99.8	39	179	1	36	2	1	66	2	2
30	I4b_S8_Klebs	Norway Outbreak (30)	Outbreak Strain	Rønning *et* al. (30)	provided as fastq	blaOXY_2-2	K. oxytoca	K. oxytoca	2	348	99.9	69	179	1	36	2	1	66	2	2
31	I4c_S9_Klebs	Norway Outbreak (30)	Outbreak Strain	Rønning *et* al. (30)	provided as fastq	blaOXY_2-2	K. oxytoca	K. oxytoca	2	348	99.9	86	179	1	36	2	1	66	2	2
32	I7_Klebs	Norway Outbreak (30)	Outbreak Strain	Rønning *et* al. (30)	provided as fastq	blaOXY_2-2	K. oxytoca	K. oxytoca	2	347	99.9	94	179	1	36	2	1	66	2	2
33	NICU-1_S10_Klebs	Norway Outbreak (30)	Control Strain	Rønning *et* al. (30)	provided as fastq	blaOXY_1-2	K. michiganensis	K. michiganensis	5	1708	99.8	77	missing	3	41	8	29	missing	6	45
34	NICU-2_Klebs	Norway Outbreak (30)	Control Strain	Rønning *et* al. (30)	provided as fastq	blaOXY_2-4	K. oxytoca	K. oxytoca	38	350	98.5	68	178	1	11	2	1	4	1	2
35	NICU-3_Klebs	Norway Outbreak (30)	Control Strain	Rønning *et* al. (30)	provided as fastq	blaOXY_6-4	K. grimontii	K. grimontii	10	1924	99.6	141	215	21	6	14	10	46	11	6
36	NICU-4_Klebs	Norway Outbreak (30)	Control Strain	Rønning *et* al. (30)	provided as fastq	blaOXY_6-5	K. grimontii	K. oxytoca	4	1931	99.8	93	467	5	6	83	75	46	8	103
37	NICU-5_Klebs	Norway Outbreak (30)	Control Strain	Rønning *et* al. (30)	provided as fastq	blaOXY_6-1	K. grimontii	K. oxytoca	5	1971	99.8	164	168	15	6	39	10	46	24	6
38	E1a_S4_Klebs	Norway Outbreak (30)	Control Strain	Rønning *et* al. (30)	provided as fastq	blaOXY_1-1	K. michiganensis	K. oxytoca	142	1817	94.4	20	202	3	5	21	13	74	6	12
39	E1b_S18_Klebs	Norway Outbreak (30)	Control Strain	Rønning *et* al. (30)	provided as fastq	blaOXY_1-1	K. michiganensis	K. oxytoca	6	1728	99.8	56	202	3	5	21	13	74	6	12
40	NZ_AP014951	Norway Outbreak Reference	Reference	SAMD00039250	download NCBI	blaOXY_6-2	K. grimontii	na	9	1962	99.6	na	263	6	6	26	10	46	24	33
41	O1-S10-66_Patient2	Graz Outbreak 2010 (4)	Outbreak Strain	SAMN39061537	newly sequenced	blaOXY_2-2	K. oxytoca	K. oxytoca	5	377	99.8	131	199	2	2	2	3	19	2	2
42	O1-S10-67_Patient1	Graz Outbreak 2010 (4)	Outbreak Strain	SAMN39061538	newly sequenced	blaOXY_2-2	K. oxytoca	K. oxytoca	5	376	99.8	130	199	2	2	2	3	19	2	2
43	O1-S10-68_Patient3	Graz Outbreak 2010 (4)	Outbreak Strain	SAMN39061539	newly sequenced	blaOXY_2-2	K. oxytoca	K. oxytoca	6	377	99.8	148	199	2	2	2	3	19	2	2
44	O1-S10-74_Patient4	Graz Outbreak 2010 (4)	Outbreak Strain	SAMN39061540	newly sequenced	blaOXY_2-2	K. oxytoca	K. oxytoca	5	380	99.8	154	199	2	2	2	3	19	2	2
45	O1-S11-17_Patient5	Graz Outbreak 2010 (4)	Outbreak Strain	SAMN39061541	newly sequenced	blaOXY_2-2	K. oxytoca	K. oxytoca	5	380	99.8	157	199	2	2	2	3	19	2	2
46	O1-33_Control1	Graz Outbreak 2010 (4)	Control Strain	SAMN39061542	newly sequenced	blaOXY_2-2	K. oxytoca	K. oxytoca	3	386	99.9	177	199	2	2	2	3	19	2	2
47	O1-S11-55_Control2	Graz Outbreak 2010 (4)	Control Strain	SAMN39061543	newly sequenced	blaOXY_2-7	K. oxytoca	K. oxytoca	2	359	99.9	121	199	2	2	2	3	19	2	2
48	O2_Patient10_K10	Graz Outbreak 2013 (7)	Outbreak Strain	SAMN39061544	newly sequenced	blaOXY_2-2	K. oxytoca	K. oxytoca	5	385	99.8	154	199	2	2	2	3	19	2	2
49	O2_Patient11_K11	Graz Outbreak 2013 (7)	Outbreak Strain	SAMN39061545	newly sequenced	blaOXY_2-2	K. oxytoca	K. oxytoca	5	381	99.8	154	199	2	2	2	3	19	2	2
50	O2_Patient12_K12	Graz Outbreak 2013 (7)	Outbreak Strain	SAMN39061546	newly sequenced	blaOXY_2-2	K. oxytoca	K. oxytoca	13	384	99.5	58	199	2	2	2	3	19	2	2
51	O2_Patient1_K1	Graz Outbreak 2013 (7)	Outbreak Strain	SAMN39061547	newly sequenced	blaOXY_2-2	K. oxytoca	K. oxytoca	5	378	99.8	161	199	2	2	2	3	19	2	2
52	O2_Patient3_K3	Graz Outbreak 2013 (7)	Outbreak Strain	SAMN39061548	newly sequenced	blaOXY_2-2	K. oxytoca	K. oxytoca	9	376	99.6	158	199	2	2	2	3	19	2	2
53	O2_Patient5_K5	Graz Outbreak 2013 (7)	Outbreak Strain	SAMN39061549	newly sequenced	blaOXY_2-2	K. oxytoca	K. oxytoca	6	377	99.8	157	199	2	2	2	3	19	2	2
54	O2_Patient9_K9	Graz Outbreak 2013 (7)	Outbreak Strain	SAMN39061550	newly sequenced	blaOXY_2-2	K. oxytoca	K. oxytoca	5	377	99.8	145	199	2	2	2	3	19	2	2
55	O2_Sinks19_Koxy13	Graz Outbreak 2013 (7)	Outbreak Strain	SAMN39061551	newly sequenced	blaOXY_2-2	K. oxytoca	K. oxytoca	8	382	99.7	150	199	2	2	2	3	19	2	2
56	O2_Sinks27_Koxy14	Graz Outbreak 2013 (7)	Outbreak Strain	SAMN39061552	newly sequenced	blaOXY_2-2	K. oxytoca	K. oxytoca	6	378	99.8	148	199	2	2	2	3	19	2	2
57	O2_Sinks39_Koxy16	Graz Outbreak 2013 (7)	Outbreak Strain	SAMN39061553	newly sequenced	blaOXY_2-2	K. oxytoca	K. oxytoca	7	384	99.7	90	199	2	2	2	3	19	2	2
58	O2_Sinks53_Koxy17	Graz Outbreak 2013 (7)	Outbreak Strain	SAMN39061554	newly sequenced	blaOXY_2-2	K. oxytoca	K. oxytoca	5	378	99.8	159	199	2	2	2	3	19	2	2
59	O2_Control217	Graz Outbreak 2013 (7)	Control Strain	SAMN39061555	newly sequenced	blaOXY_2-7	K. oxytoca	K. oxytoca	2	341	99.9	180	199	2	2	2	3	19	2	2
60	O2_Control353	Graz Outbreak 2013 (7)	Control Strain	SAMN39061556	newly sequenced	blaOXY_2-2	K. oxytoca	K. oxytoca	8	380	99.7	158	199	2	2	2	3	19	2	2
61	O2_Control402	Graz Outbreak 2013 (7)	Control Strain	SAMN39061557	newly sequenced	blaOXY_2-2	K. oxytoca	K. oxytoca	5	381	99.8	162	199	2	2	2	3	19	2	2
62	Sb-24	NCBI	-	SAMN18746951	download NCBI	blaOXY_4-3	K. pasteurii	na	17	2042	99.3	313	322	17	23	38	39	26	25	22
63	SS141 NZ_CP044527 NZ_CP04	NCBI	-	SAMN10462700	download NCBI	blaOXY_6-4	K. grimontii	na	6	1933	99.8	136	missing	21	6	14	10	missing	11	6
64	Kox205	NCBI	-	SAMN23286136	download NCBI	blaOXY_4-1	K. pasteurii	na	3	2041	99.9	42	300	13	23	38	39	69	25	22
65	RHBSTW-00054 CP055412 CP	NCBI	-	SAMN15148463	download NCBI	blaOXY_6-4	K. grimontii	na	31	1930	98.8	94	215	21	6	14	10	46	11	6
