# Supplementary material for: Core genome multilocus sequence typing (cgMLST) applicable to the monophyletic Klebsiella oxytoca species complex
Source: J Clin Microbiol. 2024 May 23;62(6):e01725-23. doi: 10.1128/jcm.01725-23 (PMC11237601; doi:10.1128/jcm.01725-23)
Supplement: Table S3 — All isolates and their respective results regarding antibiotic resistance and plasmids. [file jcm.01725-23-s0003.pdf]

Supplementary Table 3: Table containing all examined isolates included in our study and the associated results of resistance. 16 antibiotics of the AMR search in SeqSphere+ only are listed as resistance genes were found in at least one of our isolates. Also, the number of found plasmids is noted.

No.	Sample ID	Association (ref)	Gentamicin	Kanamycin	Streptomycin	Beta-lactam	Carbapenem	Cephalosporin	Erythromycin	Telithromycin	Tylosin	Chloramphenicol	Florfenicol	Phenicol	Quinolone	Sulfonamide	Tetracycline	Trimethoprim	Plasmids Found
1	#306_neg	Graz strain collection		aph(3')-Ia				blaOXY-1-1 (ESBL)						oqxA / oqxB	oqxA / oqxB				2
2	#75_neg	Graz strain collection		aph(3')-Ia				blaOXY-1-1 (ESBL)						oqxA / oqxB	oqxA / oqxB				0
3	15_neg	Graz strain collection						blaOXY-5-2 (ESBL)											3
4	25_pos	Graz strain collection						blaOXY-6-1 (ESBL)						oqxA / oqxB	oqxA / oqxB				9
5	#119_pos	Graz strain collection		aph(3')-Ia				blaOXY (ESBL)						oqxA / oqxB	oqxA / oqxB				7
6	#21_neg	Graz strain collection						blaOXY-1-9 (ESBL)						oqxB	oqxB				2
7	#19_neg	Graz strain collection						blaOXY-2-21 (ESBL)						oqxA / oqxB	oqxA / oqxB				3
8	#311_pos	Graz strain collection						blaOXY-6-2 (ESBL)						oqxA / oqxB	oqxA / oqxB				2
9	#335_pos	Graz strain collection						blaOXY-4-1 (ESBL)						oqxA / oqxB	oqxA / oqxB				1
10	21_neg	Graz strain collection						blaOXY-5-5 (ESBL)						oqxA / oqxB	oqxA / oqxB				0
11	#118_pos	Graz strain collection						blaOXY-2-1 (ESBL)						oqxA / oqxB	oqxA / oqxB				0
12	#218_neg	Graz strain collection						blaOXY-2-21 (ESBL)						oqxA / oqxB	oqxA / oqxB				3
13	S10_Klebs	Norway Outbreak (30)						blaOXY-2-2 (ESBL)						oqxA / oqxB	oqxA / oqxB				0
14	S11_Klebs	Norway Outbreak (30)						blaOXY-2-2 (ESBL)						oqxA / oqxB	oqxA / oqxB				0
15	S12_Klebs	Norway Outbreak (30)						blaOXY-2-2 (ESBL)						oqxA / oqxB	oqxA / oqxB				5
16	S13_Klebs	Norway Outbreak (30)						blaOXY-2-2 (ESBL)						oqxA / oqxB	oqxA / oqxB				0
17	S14_Klebs	Norway Outbreak (30)						blaOXY-2-2 (ESBL)						oqxA / oqxB	oqxA / oqxB				0
18	S15_Klebs	Norway Outbreak (30)						blaOXY-2-2 (ESBL)						oqxA / oqxB	oqxA / oqxB				0
19	S16_Klebs	Norway Outbreak (30)						blaOXY-2-2 (ESBL)						oqxA / oqxB	oqxA / oqxB				0
20	S17_Klebs	Norway Outbreak (30)						blaOXY-2-2 (ESBL)						oqxA / oqxB	oqxA / oqxB				0
21	S18_Klebs	Norway Outbreak (30)						blaOXY-2-2 (ESBL)						oqxA / oqxB	oqxA / oqxB				0
22	S19_Klebs	Norway Outbreak (30)						blaOXY-2-2 (ESBL)						oqxA / oqxB	oqxA / oqxB				0
23	S1_S12_Klebs	Norway Outbreak (30)						blaOXY-2-2 (ESBL)						oqxB	oqxB				0
24	S2_S11_Klebs	Norway Outbreak (30)						blaOXY-2-2 (ESBL)						oqxA / oqxB	oqxA / oqxB				0
25	S6_Klebs	Norway Outbreak (30)						blaOXY-2-2 (ESBL)						oqxA / oqxB	oqxA / oqxB				0
26	S7_Klebs	Norway Outbreak (30)						blaOXY-2-2 (ESBL)						oqxA / oqxB	oqxA / oqxB				0
27	S8_Klebs	Norway Outbreak (30)						blaOXY-2-2 (ESBL)						oqxA / oqxB	oqxA / oqxB				0
28	S9_Klebs	Norway Outbreak (30)						blaOXY-2-2 (ESBL)						oqxA / oqxB	oqxA / oqxB				0
29	I4a_S7_Klebs	Norway Outbreak (30)						blaOXY-2-2 (ESBL)						oqxA / oqxB	oqxA / oqxB				0
30	I4b_S8_Klebs	Norway Outbreak (30)						blaOXY-2-2 (ESBL)						oqxA / oqxB	oqxA / oqxB				0
31	I4c_S9_Klebs	Norway Outbreak (30)						blaOXY-2-2 (ESBL)						oqxA / oqxB	oqxA / oqxB				0
32	I7_Klebs	Norway Outbreak (30)						blaOXY-2-2 (ESBL)						oqxA / oqxB	oqxA / oqxB				0
33	NICU-1_S10_Klebs	Norway Outbreak (30)						blaOXY-1-2 (ESBL)						oqxA / oqxB	oqxA / oqxB				7
34	NICU-2_Klebs	Norway Outbreak (30)						blaOXY-2-4 (ESBL)						oqxA / oqxB	oqxA / oqxB				4
35	NICU-3_Klebs	Norway Outbreak (30)						blaOXY-6-4 (ESBL)						oqxA / oqxB	oqxA / oqxB				3
36	NICU-4_Klebs	Norway Outbreak (30)						blaOXY-6-5 (ESBL)						oqxA / oqxB	oqxA / oqxB				0
37	NICU-5_Klebs	Norway Outbreak (30)						blaOXY-6-1 (ESBL)						oqxA / oqxB	oqxA / oqxB				2
38	E1a_S4_Klebs	Norway Outbreak (30)		aph(3')-Ia				blaOXY-1-1 (ESBL)						oqxA / oqxB	oqxA / oqxB				1
39	E1b_S18_Klebs	Norway Outbreak (30)		aph(3')-Ia				blaOXY-1-1 (ESBL)						oqxA / oqxB	oqxA / oqxB				1
40	NZ_AP014951_plasmids	Norway Outbreak Reference						blaOXY-6-2 (ESBL)						oqxA / oqxB	oqxA / oqxB				4
41	O1-S10-66_Patient2	Graz Outbreak 2010 (4)	aac(6')-Ib'		aadA1	blaOXA / blaTEM-1	blaKPC-2 (ca	blaOXY-2-2 (ESBL)				floR	floR	oqxA / oqxB	oqxA / oqxB	sul1 / sul2	tet(E)	dfrB1	5
42	O1-S10-67_Patient1	Graz Outbreak 2010 (4)	aac(6')-Ib'		aadA1	blaOXA / blaTEM-1	blaKPC-2 (ca	blaOXY-2-2 (ESBL)				floR	floR	oqxA / oqxB	oqxA / oqxB	sul1 / sul2	tet(E)	dfrB1	5
43	O1-S10-68_Patient3	Graz Outbreak 2010 (4)	aac(6')-Ib'		aadA1	blaOXA / blaTEM-1	blaKPC-2 (ca	blaOXY-2-2 (ESBL)				floR	floR	oqxA / oqxB	oqxA / oqxB	sul1 / sul2	tet(E)	dfrB1	5
44	O1-S10-74_Patient4	Graz Outbreak 2010 (4)	aac(6')-Ib'		aadA1	blaOXA / blaTEM-1	blaKPC-2 (ca	blaOXY-2-2 (ESBL)				floR	floR	oqxA / oqxB	oqxA / oqxB	sul1 / sul2	tet(E)		5
45	O1-S11-17_Patient5	Graz Outbreak 2010 (4)	aac(6')-Ib'		aadA1	blaOXA / blaTEM-1	blaKPC-2 (ca	blaOXY-2-2 (ESBL)				floR	floR	oqxA / oqxB	oqxA / oqxB	sul1 / sul2	tet(E)	dfrB1	4
46	O1-33_Control1	Graz Outbreak 2010 (4)						blaOXY-2-2 (ESBL)						oqxA / oqxB	oqxA / oqxB				0
47	O1-S11-55_Control2	Graz Outbreak 2010 (4)						blaOXY-2-7 (ESBL)						oqxA / oqxB	oqxA / oqxB				1
48	O2_Patient10_K10	Graz Outbreak 2013 (7)	aac(6')-Ib'			blaOXA / blaTEM-1	blaKPC-2 (ca	blaOXY-2-2 (ESBL)				floR	floR	oqxA / oqxB	oqxA / oqxB	sul1 / sul2	tet(E)		4
49	O2_Patient11_K11	Graz Outbreak 2013 (7)	aac(6')-Ib'		aadA1	blaOXA / blaTEM-1	blaKPC-2 (ca	blaOXY-2-2 (ESBL)				floR	floR	oqxA / oqxB	oqxA / oqxB	sul1 / sul2	tet(E)	dfrB1	6
50	O2_Patient12_K12	Graz Outbreak 2013 (7)	aac(6')-Ib'		aadA1	blaOXA / blaTEM-1	blaKPC-2 (ca	blaOXY-2-2 (ESBL)				floR	floR	oqxA / oqxB	oqxA / oqxB	sul1 / sul2	tet(E)		5
51	O2_Patient1_K1	Graz Outbreak 2013 (7)	aac(6')-Ib'			blaOXA / blaTEM-1	blaKPC-2 (ca	blaOXY-2-2 (ESBL)				floR	floR	oqxA / oqxB	oqxA / oqxB	sul1 / sul2	tet(E)		3
52	O2_Patient3_K3	Graz Outbreak 2013 (7)	aac(6')-Ib'			blaOXA / blaTEM-1	blaKPC-2 (ca	blaOXY-2-2 (ESBL)				floR	floR	oqxA / oqxB	oqxA / oqxB	sul1 / sul2	tet(E)		4
53	O2_Patient5_K5	Graz Outbreak 2013 (7)	aac(6')-Ib'		aadA1	blaOXA / blaTEM-1	blaKPC-2 (ca	blaOXY-2-2 (ESBL)				floR	floR	oqxA / oqxB	oqxA / oqxB	sul1 / sul2	tet(E)		5
54	O2_Patient9_K9	Graz Outbreak 2013 (7)	aac(6')-Ib'			blaOXA / blaTEM-1	blaKPC-2 (ca	blaOXY-2-2 (ESBL)				floR	floR	oqxA / oqxB	oqxA / oqxB	sul1 / sul2	tet(E)		3
55	O2_Sinks19_Koxy13	Graz Outbreak 2013 (7)	aac(6')-Ib'		aadA1	blaOXA / blaTEM-1	blaKPC-2 (ca	blaOXY-2-2 (ESBL)				floR	floR	oqxA / oqxB	oqxA / oqxB	sul1 / sul2	tet(E)		4
56	O2_Sinks27_Koxy14	Graz Outbreak 2013 (7)	aac(6')-Ib'			blaOXA / blaTEM-1	blaKPC-2 (ca	blaOXY-2-2 (ESBL)				floR	floR	oqxA / oqxB	oqxA / oqxB	sul1 / sul2	tet(E)		5
57	O2_Sinks39_Koxy16	Graz Outbreak 2013 (7)	aac(6')-Ib'		aadA1	blaOXA / blaTEM-1	blaKPC-2 (ca	blaOXY-2-2 (ESBL)				floR	floR	oqxA / oqxB	oqxA / oqxB	sul1 / sul2	tet(E)		4
58	O2_Sinks53_Koxy17	Graz Outbreak 2013 (7)	aac(6')-Ib'		aadA1	blaOXA / blaTEM-1	blaKPC-2 (ca	blaOXY-2-2 (ESBL)				floR	floR	oqxA / oqxB	oqxA / oqxB	sul1 / sul2	tet(E)		3
59	O2_Control217	Graz Outbreak 2013 (7)						blaOXY-2-7 (ESBL)						oqxA / oqxB	oqxA / oqxB				0
60	O2_Control353	Graz Outbreak 2013 (7)						blaOXY-2-2 (ESBL)						oqxA / oqxB	oqxA / oqxB				0
61	O2_Control402	Graz Outbreak 2013 (7)	aac(6')-Ib'		aadA1			blaOXY-2-2 (ESBL)				floR	floR	oqxA / oqxB	oqxA / oqxB	sul1 / sul2	tet(E)	dfrB1	4
62	Sb-24	NCBI Download						blaOXY-4-3 (ESBL)						oqxA	oqxA				1
63	SS141 NZ_CP044527 NZ_CP04	NCBI Download						blaOXY-6-4 (ESBL)						oqxA / oqxB	oqxA / oqxB				3
64	Kox205	NCBI Download	aac(3)-IId / aa		aph(3'')-Ib	blaTEM-1	blaIMP-4	blaOXY-4-1 (ESBL) / blaSI	mph(A)	mph(A)	mph(A)	catA2 / catB3		oqxA / oqxB	oqxA / oqxB / qr	sul1, sul1 / sul2		dfrA14	3
65	RHBSTW-00054 CP055412 CP0	NCBI Download						blaOXY-6-4 (ESBL)						oqxA / oqxB	oqxA / oqxB				5
