# Supplementary material for: Core genome multilocus sequence typing (cgMLST) applicable to the monophyletic Klebsiella oxytoca species complex
Source: J Clin Microbiol. 2024 May 23;62(6):e01725-23. doi: 10.1128/jcm.01725-23 (PMC11237601; doi:10.1128/jcm.01725-23)
Supplement: Table S4 — All isolates and their respective results regarding the til gene cluster. [file jcm.01725-23-s0004.pdf]

Supplementary Table 4: A list of all examined isolates included in our study with results of the toxigenic *til* cluster. Included are the respective Sample ID used also in figures, the species assignment from the blaOXY gene and the association. The number of missing alleles of the tilivalline cluster gives the number of missing numbers for the 12 genes in the following columns and was used to assign "yes", "no" or "yes (xxx missing)" in the column od toxicity based on genetic results. The cutoff for confirmed toxicity was more than 3 missing alleles.

No.	Species blaOXY	Sample ID	Association	ST	toxicity based on genetic results	Number of missing alleles of tilivalline cluster	NDX50_24820 npsB	NDX50_24830 npsA	NDX50_24835 aroX	NDX50_24840 dhbX	NDX50_24845 icmX	NDX50_24850 adsX	NDX50_24855 hmoX (knockout also pos)	NDX50_24865 uvrX (immunity gene)	NDX50_24870 mfsX (immunity gene)	NDX50_24810 marR	NDX50_24815 npsC	NDX50_24825 tdhA (very small)
0	Klebsiella oxytoca	Reference - AHC-6	Reference *til* gene cluster	1	yes	0	7	7	8	6	7	8	1	11	11	6	6	4
1	Klebsiella michiganensis	#306_neg	Graz strain collection	27	no	12	(not found)	(not found)	(not found)	(not found)	(not found)	(not found)	(not found)	(not found)	(not found)	(not found)	(not found)	(not found)
2	Klebsiella michiganensis	#75_neg	Graz strain collection	11	no	12	(not found)	(not found)	(not found)	(not found)	(not found)	(not found)	(not found)	(not found)	(not found)	(not found)	(not found)	(not found)
3	Klebsiella michiganensis	15_neg	Graz strain collection	82	no	12	(not found)	(not found)	(not found)	(not found)	(not found)	(not found)	(not found)	(not found)	(not found)	(not found)	(not found)	(not found)
4	Klebsiella grimontii	25_pos	Graz strain collection	377	yes (hmoX missing)	1	11	11	12	8	10	12	(not found)	15	13	7	9	1
5	Klebsiella michiganensis	#119_pos	Graz strain collection	35	yes (Small gene + hmoX missing)	2	4	3	4	4	4	4	(not found)	6	7	4	(not found)	3
6	Klebsiella michiganensis	#21_neg	Graz strain collection	40	no	12	(not found)	(not found)	(not found)	(not found)	(not found)	(not found)	(not found)	(not found)	(not found)	(not found)	(not found)	(not found)
7	Klebsiella oxytoca	#19_neg	Graz strain collection	78	no	8	(not found)	(not found)	(not found)	(not found)	(not found)	(not found)	(not found)	2	2	2	2	(not found)
8	Klebsiella grimontii	#311_pos	Graz strain collection	216	yes (hmoX missing)	1	1	1	1	1	1	1	(not found)	3	3	3	3	1
9	Klebsiella pasteurii	#335_pos	Graz strain collection	?	yes (Small gene + hmoX missing)	2	12	12	13	9	11	13	(not found)	16	14	9	(not found)	5
10	Klebsiella michiganensis	21_neg	Graz strain collection	80	no	12	(not found)	(not found)	(not found)	(not found)	(not found)	(not found)	(not found)	(not found)	(not found)	(not found)	(not found)	(not found)
11	Klebsiella oxytoca	#118_pos	Graz strain collection	19	yes	0	2	2	2	2	2	2	3	4	5	2	1	2
12	Klebsiella oxytoca	#218_neg	Graz strain collection	58	no	8	(not found)	(not found)	(not found)	(not found)	(not found)	(not found)	(not found)	2	4	2	4	(not found)
13	Klebsiella oxytoca	S10_Klebs	Norway Outbreak (30)	179	yes	0	3	2	3	3	3	3	2	5	6	1	1	2
14	Klebsiella oxytoca	S11_Klebs	Norway Outbreak (30)	179	yes	0	3	2	3	3	3	3	2	5	6	1	1	2
15	Klebsiella oxytoca	S12_Klebs	Norway Outbreak (30)	179	yes	0	3	2	3	3	3	3	2	5	6	1	1	2
16	Klebsiella oxytoca	S13_Klebs	Norway Outbreak (30)	179	yes	0	3	2	3	3	3	3	2	5	6	1	1	2
17	Klebsiella oxytoca	S14_Klebs	Norway Outbreak (30)	179	yes	0	3	2	3	3	3	3	2	5	6	1	1	2
18	Klebsiella oxytoca	S15_Klebs	Norway Outbreak (30)	179	yes	0	3	2	3	3	3	3	2	5	6	1	1	2
19	Klebsiella oxytoca	S16_Klebs	Norway Outbreak (30)	179	yes	0	3	2	3	3	3	3	2	5	6	1	1	2
20	Klebsiella oxytoca	S17_Klebs	Norway Outbreak (30)	179	yes	0	3	2	3	3	3	3	2	5	6	1	1	2
21	Klebsiella oxytoca	S18_Klebs	Norway Outbreak (30)	179	yes	0	3	2	3	3	3	3	2	5	6	1	1	2
22	Klebsiella oxytoca	S19_Klebs	Norway Outbreak (30)	179	yes	0	3	2	3	3	3	3	2	5	6	1	1	2
23	Klebsiella oxytoca	S1_S12_Klebs	Norway Outbreak (30)	179	yes	0	3	2	3	3	3	3	2	5	6	1	1	2
24	Klebsiella oxytoca	S2_S11_Klebs	Norway Outbreak (30)	179	yes	0	3	2	3	3	3	3	2	5	6	1	1	2
25	Klebsiella oxytoca	S6_Klebs	Norway Outbreak (30)	179	yes	0	3	2	3	3	3	3	2	5	6	1	1	2
26	Klebsiella oxytoca	S7_Klebs	Norway Outbreak (30)	179	yes	0	3	2	3	3	3	3	2	5	6	1	1	2
27	Klebsiella oxytoca	S8_Klebs	Norway Outbreak (30)	179	yes	0	3	2	3	3	3	3	2	5	6	1	1	2
28	Klebsiella oxytoca	S9_Klebs	Norway Outbreak (30)	179	yes	0	3	2	3	3	3	3	2	5	6	1	1	2
29	Klebsiella oxytoca	I4a_S7_Klebs	Norway Outbreak (30)	179	yes	0	3	2	3	3	3	3	2	5	6	1	1	2
30	Klebsiella oxytoca	I4b_S8_Klebs	Norway Outbreak (30)	179	yes	0	3	2	3	3	3	3	2	5	6	1	1	2
31	Klebsiella oxytoca	I4c_S9_Klebs	Norway Outbreak (30)	179	yes	0	3	2	3	3	3	3	2	5	6	1	1	2
32	Klebsiella oxytoca	I7_Klebs	Norway Outbreak (30)	179	yes	0	3	2	3	3	3	3	2	5	6	1	1	2
33	Klebsiella michiganensis	NICU-1_S10_Klebs	Norway Outbreak (30)	missing	no	12	(not found)	(not found)	(not found)	(not found)	(not found)	(not found)	(not found)	(not found)	(not found)	(not found)	(not found)	(not found)
34	Klebsiella oxytoca	NICU-2_Klebs	Norway Outbreak (30)	178	yes	0	5	5	6	3	3	6	4	9	9	6	5	2
35	Klebsiella grimontii	NICU-3_Klebs	Norway Outbreak (30)	215	yes (hmoX missing)	1	8	8	9	7	1	9	(not found)	12	12	8	7	1
36	Klebsiella grimontii	NICU-4_Klebs	Norway Outbreak (30)	467	yes (hmoX missing)	1	10	10	11	1	9	11	(not found)	14	12	7	8	1
37	Klebsiella grimontii	NICU-5_Klebs	Norway Outbreak (30)	168	yes (hmoX missing)	1	9	9	10	1	8	10	(not found)	13	3	3	3	1
38	Klebsiella michiganensis	E1a_S4_Klebs	Norway Outbreak (30)	202	no	12	(not found)	(not found)	(not found)	(not found)	(not found)	(not found)	(not found)	(not found)	(not found)	(not found)	(not found)	(not found)
39	Klebsiella michiganensis	E1b_S18_Klebs	Norway Outbreak (30)	202	no	12	(not found)	(not found)	(not found)	(not found)	(not found)	(not found)	(not found)	(not found)	(not found)	(not found)	(not found)	(not found)
40	Klebsiella grimontii	NZ_AP014951	Norway Outbreak Reference	263	yes (hmoX missing)	1	6	6	7	5	6	7	(not found)	10	10	7	3	1
41	Klebsiella oxytoca	O1-S10-66_Patient2	Graz Outbreak 2010 (4)	199	no	8	(not found)	(not found)	(not found)	(not found)	(not found)	(not found)	(not found)	1	1	1	1	(not found)
42	Klebsiella oxytoca	O1-S10-67_Patient1	Graz Outbreak 2010 (4)	199	no	8	(not found)	(not found)	(not found)	(not found)	(not found)	(not found)	(not found)	1	1	1	1	(not found)
43	Klebsiella oxytoca	O1-S10-68_Patient3	Graz Outbreak 2010 (4)	199	no	8	(not found)	(not found)	(not found)	(not found)	(not found)	(not found)	(not found)	1	1	1	1	(not found)
44	Klebsiella oxytoca	O1-S10-74_Patient4	Graz Outbreak 2010 (4)	199	no	8	(not found)	(not found)	(not found)	(not found)	(not found)	(not found)	(not found)	1	1	1	1	(not found)
45	Klebsiella oxytoca	O1-S11-17_Patient5	Graz Outbreak 2010 (4)	199	no	8	(not found)	(not found)	(not found)	(not found)	(not found)	(not found)	(not found)	1	1	1	1	(not found)
46	Klebsiella oxytoca	O1-33_Control1	Graz Outbreak 2010 (4)	199	no	8	(not found)	(not found)	(not found)	(not found)	(not found)	(not found)	(not found)	1	1	1	1	(not found)
47	Klebsiella oxytoca	O1-S11-55_Control2	Graz Outbreak 2010 (4)	199	no	8	(not found)	(not found)	(not found)	(not found)	(not found)	(not found)	(not found)	7	1	1	1	(not found)
48	Klebsiella oxytoca	O2_Patient10_K10	Graz Outbreak 2013 (7)	199	no	8	(not found)	(not found)	(not found)	(not found)	(not found)	(not found)	(not found)	1	1	1	1	(not found)
49	Klebsiella oxytoca	O2_Patient11_K11	Graz Outbreak 2013 (7)	199	no	8	(not found)	(not found)	(not found)	(not found)	(not found)	(not found)	(not found)	1	1	1	1	(not found)
50	Klebsiella oxytoca	O2_Patient12_K12	Graz Outbreak 2013 (7)	199	no	8	(not found)	(not found)	(not found)	(not found)	(not found)	(not found)	(not found)	1	1	1	1	(not found)
51	Klebsiella oxytoca	O2_Patient1_K1	Graz Outbreak 2013 (7)	199	no	8	(not found)	(not found)	(not found)	(not found)	(not found)	(not found)	(not found)	1	1	1	1	(not found)
52	Klebsiella oxytoca	O2_Patient3_K3	Graz Outbreak 2013 (7)	199	no	8	(not found)	(not found)	(not found)	(not found)	(not found)	(not found)	(not found)	1	1	1	1	(not found)
53	Klebsiella oxytoca	O2_Patient5_K5	Graz Outbreak 2013 (7)	199	no	8	(not found)	(not found)	(not found)	(not found)	(not found)	(not found)	(not found)	1	1	1	1	(not found)
54	Klebsiella oxytoca	O2_Patient9_K9	Graz Outbreak 2013 (7)	199	no	8	(not found)	(not found)	(not found)	(not found)	(not found)	(not found)	(not found)	1	1	1	1	(not found)
55	Klebsiella oxytoca	O2_Sinks19_Koxy13	Graz Outbreak 2013 (7)	199	no	8	(not found)	(not found)	(not found)	(not found)	(not found)	(not found)	(not found)	1	1	1	1	(not found)
56	Klebsiella oxytoca	O2_Sinks27_Koxy14	Graz Outbreak 2013 (7)	199	no	8	(not found)	(not found)	(not found)	(not found)	(not found)	(not found)	(not found)	1	1	1	1	(not found)
57	Klebsiella oxytoca	O2_Sinks39_Koxy16	Graz Outbreak 2013 (7)	199	no	8	(not found)	(not found)	(not found)	(not found)	(not found)	(not found)	(not found)	1	1	1	1	(not found)
58	Klebsiella oxytoca	O2_Sinks53_Koxy17	Graz Outbreak 2013 (7)	199	no	8	(not found)	(not found)	(not found)	(not found)	(not found)	(not found)	(not found)	1	1	1	1	(not found)
59	Klebsiella oxytoca	O2_Control217	Graz Outbreak 2013 (7)	199	no	8	(not found)	(not found)	(not found)	(not found)	(not found)	(not found)	(not found)	1	1	1	1	(not found)
60	Klebsiella oxytoca	O2_Control353	Graz Outbreak 2013 (7)	199	no	8	(not found)	(not found)	(not found)	(not found)	(not found)	(not found)	(not found)	1	1	1	1	(not found)
61	Klebsiella oxytoca	O2_Control402	Graz Outbreak 2013 (7)	199	no	8	(not found)	(not found)	(not found)	(not found)	(not found)	(not found)	(not found)	1	1	1	1	(not found)
62	Klebsiella pasteurii	Sb-24	NCBI Download	322	yes (Small gene + hmoX missing)	2	13	13	13	9	11	14	(not found)	17	15	9	(not found)	5
63	Klebsiella grimontii	SS141 NZ CP044527 NZ CP0	NCBI Download	missing	yes (hmoX missing)	1	15	15	9	7	1	16	(not found)	20	12	8	7	1
64	Klebsiella pasteurii	Kox205	NCBI Download	300	yes (Small gene + hmoX missing)	2	14	14	14	10	11	15	(not found)	18	15	10	(not found)	5
65	Klebsiella grimontii	RHBSTW-00054 CP055412 CP	NCBI Download	215	yes (hmoX missing)	1	15	8	15	7	1	16	(not found)	19	12	8	7	1
